# Age at Immigration and Depression in Old Age: The Mediating Role of Contemporary Relationships With Adult Children

**DOI:** 10.1093/geroni/igaa057.087

**Published:** 2020-12-16

**Authors:** Heejung Jang

**Affiliations:** University of Michigan, Ann Arbor, Michigan, United States

## Abstract

For the growing population of older immigrants in the United States, both age at immigration and familial relationships are important factors affecting psychological well-being. This study explores how age at immigration and contemporary relationships with adult children combine to explain older immigrants’ depressive symptoms. This study uses 2014 Health and Retirement Study data from a sample of 759 immigrants age 65 and older who have at least one adult child age 21 or older. A series of ordinary least squares regressions and mediational analyses were conducted. Findings indicate that two aspects of familial relationships, associational solidarity and structural solidarity, significantly mediate the association between age at immigration and depressive symptoms. Specifically, immigrating in later-life was associated with a lower level of depressive symptoms through its relationship with structural solidarity. Immigrating in later-life was also associated with a higher level of depressive symptoms through its relationship with associational solidarity. In addition, giving monetary support to children and providing care for grandchildren may alleviate depressive symptoms for older immigrants. This study suggests that relationships with adult children may differ with age at immigration. The types of support that older immigrants provide to their adult children may be crucial because such support may instill a sense of obligation and reciprocity that may be beneficial to the psychological well-being of older immigrants.

